# Influence of Blood Glycemia Levels in Refraction, Binocular Vision and Accommodation: A Case Report

**DOI:** 10.3390/reports7020022

**Published:** 2024-03-23

**Authors:** Marc Argilés, Jessica Sala-Oller, Bernat Sunyer-Grau, Cristina Rovira-Gay, Luis Pérez-Mañá

**Affiliations:** 1 School of Optics and Optometry, Universitat Politècnica de Catalunya, 08222 Terrassa, Spain; marc.argiles@upc.edu (M.A.); jsalaoller99@gmail.com (J.S.-O.); bernat.sunyer@upc.edu (B.S.-G.); 2 Centre for Sensors, Instruments and Systems Development (CD-6), Universitat Politècnica de Catalunya, 08222 Terrassa, Spain; cristina.rovira@upc.edu

**Keywords:** blood glycemia, binocular vision, ocular accommodation

## Abstract

This case report provides us with insight on how blood glycemia affects refraction, vergence and accommodation in a single diabetic patient. A 21-year-old type I diabetic woman was the subject studied in this report. Refraction, near and far fusional vergence ranges, near point of convergence, monocular accommodative facility, amplitude of accommodation, lag of accommodation, and near and far phoria, were measured before and after controlled caloric intake and insulin injection. Measurements were taken a total of 10 times, once a week for 10 consecutive weeks. Blood glycemia levels were provided by a measuring device that was attached to the patient’s body at all times. Statistically significant differences were found in the glucose levels before and after lunch, *p* = 0.041, sphere refraction of the right eye, *p* = 0.016, but not in the left eye, *p* = 0.051. Accommodative facility in both right and left eyes, *p* = 0.019, *p* = 0.028, respectively, and amplitude of accommodation, *p* = 0.016, *p* = 0.019, right and left eyes, respectively were statistically different before and after insulin injection. In a 21-year-old subject with type I diabetes, a diminution in blood glucose levels influences refractive myopic state, and is associated with a decrease in accommodative facility and in amplitude of accommodation.

## 1. Introduction

Diabetes mellitus is a metabolic disease characterized by an abnormally high concentration of glucose in the blood (hyperglycemia). It can be caused by a lack of insulin due to a malfunction of the pancreas, or by a failure of cells to respond to insulin, which is known as insulin resistance. Insulin is a hormone produced and secreted in the pancreas by beta-cells, and its function is to regulate blood sugar levels by allowing glucose to enter the cells [1]. Insufficient levels of insulin in the blood, as seen in diabetes mellitus, prevent glucose from entering cells and result in abnormally high levels of glucose in the blood. If high blood sugar levels persist, serious health problems might arise. Approximately 422 million people suffer from diabetes worldwide [2]. There are two different types of diabetes: Type I and Type II. Type I is present in approximately 10% of the population, the age of onset is usually under forty years old, and is caused by an autoimmune reaction in which the organism attacks the beta cells in the pancreas. Type II diabetes represents 90% of diabetes cases and normally appears in subjects between 35 and 40 years old [3].

Diabetes, and specifically hyperglycemia, are associated with a myopic shift in refraction [4,5]. Although extensively studied, these changes in refraction are not completely clear nor fully understood. Some studies have found no change in refractive error between normal blood glucose levels and hyperglycemia [6], even though in one individual high blood glucose levels were found to be associated with changes in anterior lens surface and changes in the refractive index of the lens. Moreover, type I diabetes is associated with a lower amplitude of accommodation compared to healthy age-matched controls. In one study not only was amplitude of accommodation lower in patients with type I diabetes (*n* = 43) than in controls (*n* = 32), but also a loss of amplitude of accommodation strongly correlated with the duration of the disease [7]. Other authors obtained similar results in a population of diabetic schoolchildren who had reduced amplitude of accommodation compared to their healthy counterparts [8]. The goal of this study is to better understand how glycemia levels influence refraction, binocular vision, and accommodation in a 21-year-old type I diabetic woman.

## 2. Case Presentation Section

A 21-year-old girl diagnosed at age 4 with type I diabetes mellitus is the subject studied in this clinical case report. The study was approved by an institutional review board (UPC), and written consent information was obtained for identifiable health information included in this case report. The patient regularly wears a device that measures blood glucose levels using the FreeStyle Libre Link System, an automatic system that measures blood sugar levels and displays the results on a mobile phone application [9].

Refractive state, binocular vision, and accommodation were evaluated 10 times, once a week for 10 consecutive weeks, before and after lunch in all 10 days. Caloric intake was controlled for all 10 meals. Lunch on all measurement days consisted of grilled chicken breast, rice, and broccoli in equal amounts. Measurements were taken at 1 pm and 3 pm, before and after lunch, respectively, in the same room and under the same conditions from an optometrist with high experience in the visual examination who was masked from the aim of the current study in order to avoid a possible bias in the measures. Glucose levels were recorded before and after the visual examination. All measurements were conducted once per week. Pre-lunch values were grouped into “pre”, and post-lunch values into “post”. The daily amount of insulin injected every day of measurement before lunch was 3 mL. Refraction (spherical and cylindrical power) was measured with an autorefractometer (Topcon, KR-800A, Tokyo 174-8580, Japan). Fusional vergence ranges (convergence and divergence) were measured using a Risley prism (phoropter) at distance (6 m) and near (40 cm), using a 20/30 visual acuity letter column as a target. The near point of convergence was tested with an RAF rule, monocular accommodative facility was assessed with a mounted flipper (±2.00 D) at 40 cm, and monocular amplitude of accommodation using minus lenses (in phoropter at 40 cm) using a 20/30 visual acuity letter column as a target. Error of accommodation was measured with the monocular estimated method (MEM) at near (0.4 m), and dissociated phoria was measured with the Von Graefe technique at either near (0.4 m) and far (0.6 m).

**Figure 1:**
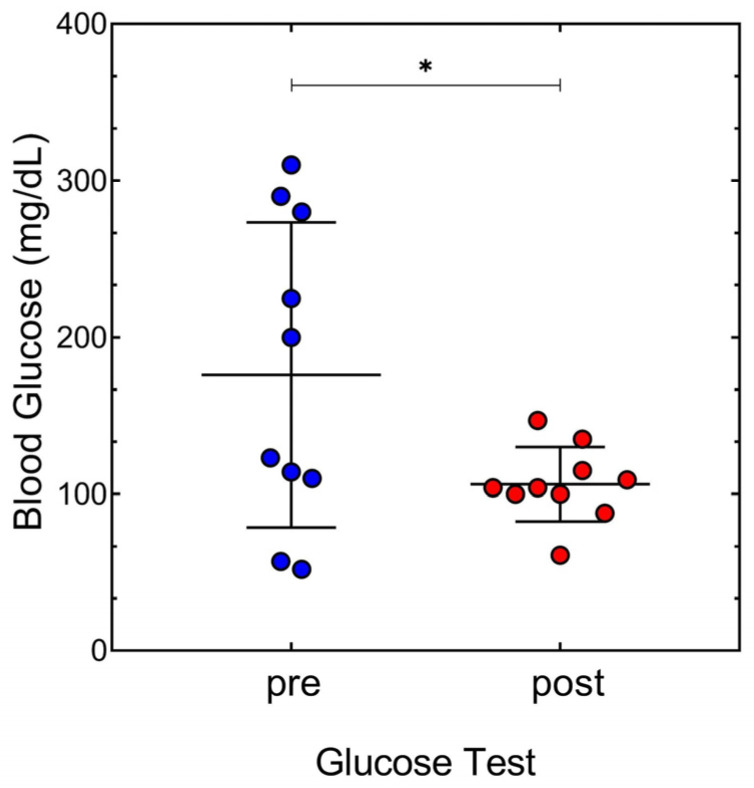
Scatter plot with the median and standard deviation of glucose levels before “pre”, and after “post” lunch. Concentration of glucose in the blood is expressed in mg/dL. Every circle indicates each day of measures (n = 10). We can see that the variance in “pre” lunch is higher than “post” lunch. Coefficient of variation in “pre” is 55.28% and 22.26% in “post”. * indicates statistically significant differences between the tests (*p* value < 0.05).

The patient’s habitual refractive correction was −0.75 −0.50 to 90° and −1.25 −0.50 to 90° in the right and left eyes, respectively. Average “pre” and “post” results and *p* values of all visual parameters studied are presented in Table 1. A one-way ANOVA was computed to study the possible differences in visual status. Glucose levels were higher before (176.10 ± 97.34 mg/dL) than after lunch (106.30 ± 23.66 mg/dL), and statistically different, F(1,18) = 4.85, *p* = 0.041, Figure 1.

**Figure 2:**
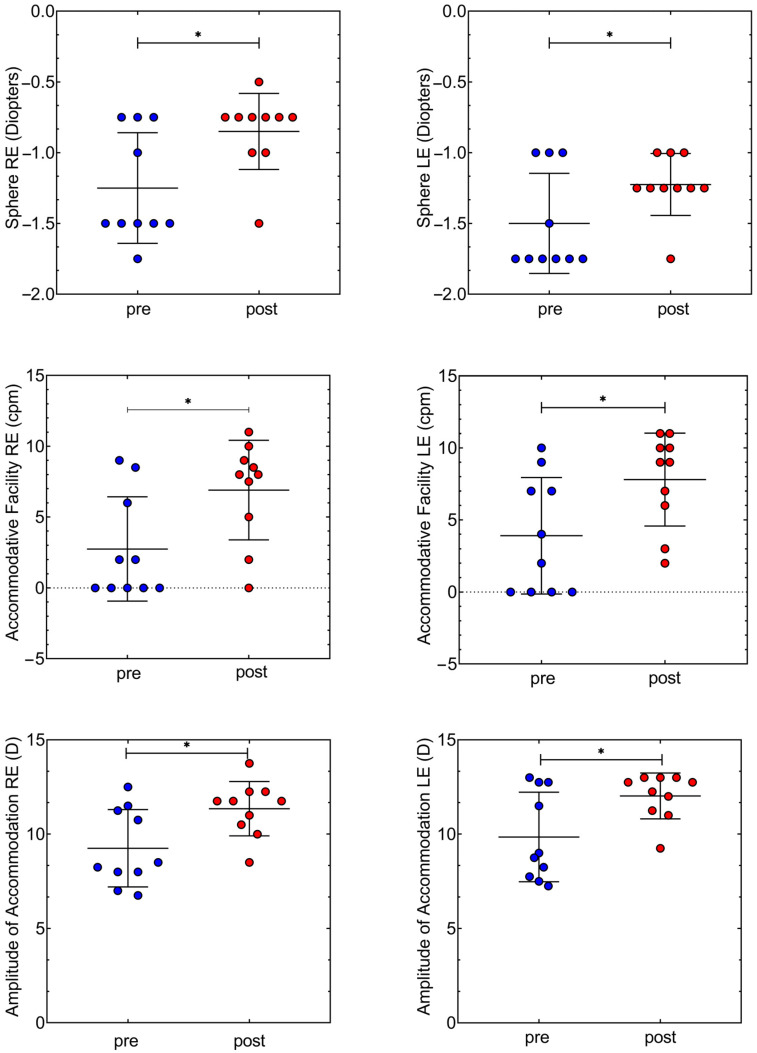
Scatter plot with the median and standard deviation “pre” and “post” measures in objective refractive state of the sphere (first two graphs), accommodative facility (two middle graphs) and amplitude of accommodation (two last graphs). Graphs presented on the left side of the page correspond to values of the right eye (RE) whereas graphs on the right side of the page correspond to values of the left eye (LE). In the objective refractive state (sphere) coefficient of variation in “pre” and “post” measures for the right eye is very similar, 31.27% and 31.62%, respectively. The same trend can be observed for the left eye in “pre”, 23.57% and “post”, 17.87%. Coefficient of variation in “pre” and “post” measures for the accommodative facility in the right eye is very different in “pre”, 133.7% than “post”, 50.99%. Similar variation is observed for the left eye,103.6% and 41.34%, respectively. In amplitude of accommodation, the coefficient of variation is found to be very similar between “pre” and “post” in the right eye, from 22.18% to 12.68%, and in the left eye, from 24.08% to 10.11%. (*) indicates statistically significant differences between the tests (*p* value < 0.05).

Statistically significant differences were found in the right eye, F(1,18) = 7.11, *p* = 0.016, but not in the left eye, F(1,18) = 4.37, *p* = 0.051, between pre-post lunch, Figure 2. Amplitude of accommodation was lower before lunch in both right and left eyes, 9.85 ± 2.37 D, 9.85 ± 12.02 D, respectively, than after, 11.35 ± 1.44 D, 12.02 ± 1.21 D, respectively. In both eyes there was a statistically significant difference in accommodative facility values before and after eating, in the right eye we found a change from 2.75 ± 3.67 cycles per minute (cpm) to 6.90 ± 3.51 cpm, F(1,18) = 6.65, *p* = 0.019 and in the left eye from 3.90 ± 4.04 cpm to 7.80 ± 3.22 cpm, F(1,18) = 5.69, *p* = 0.028. Accommodative facility cycles per minute doubled after caloric intake in both eyes. No statistically significant differences were found between “pre” and “post” results in cylindrical power in refraction, fusional vergence amplitudes, near point of convergence, MEM, and dissociated phoria.

## 3. Discussion

This clinical case demonstrates how refractive state and binocular and accommodative systems can vary depending on blood sugar levels in a 21 years old subject with type I diabetes. The patient ingested the same number of calories in all meals to ensure reliable and repetitive data. Glucose levels were statistically significant before and after lunch. Therefore, optometric data could be compared between “pre” and “post” values after lunch and insulin injection, which is usually done in many patients with diabetes type I. Consistent with previous studies [10,11,12,13] we have found refraction to undergo a hyperopic change after normalization of blood sugar levels, and agreed that a decrease in blood glucose cause a diminution of myopia refraction, as we can see in Table 1 with the myopic change in right and left eye. In other words, hyperglycemia is related to excessively myopic refraction. More importantly, the monocular amplitude of accommodation and the monocular accommodative facility were reduced before lunch, and when blood glycemia was reduced after lunch, the accommodative facility nearly doubled the values for right and left eye (Table 1). This variation could be explained due to a reduction of refractive index in the crystalline [14], or by corneal topographic parameters changes [11]. The rest of the optometric tests evaluated (Table 1) did not show statistically significant differences.

Lin et al. (2009) found hyperopic shift during glucose reduction to be not complemented with variations in axial length, lens thickness or accommodation amplitude [13]. Also, in 2014, low accommodation amplitude was found in type 1 diabetes adults compared to a non diabetic group [7]. Other authors, however, have found lower accommodation amplitude and greater accommodative lag in black Africans with type I diabetes compared to sex and age matched controls [15]. Contrary to these publications, in the present study no variation was found in the mean lag of accommodation after the restoration of blood sugar levels. Moreover, recent findings of a cross-sectional study found that accommodative disorders are linked to type 1 diabetes [16]. In this clinical case, we showed that fluctuations in glycemic levels influence the accommodative system more than the vergence system, without changes in near point of convergence, near and far phorias, and fusional vergence amplitudes, compared with amplitude of accommodation and accommodative facility. However, all ocular measures returned to the normal state after the proper blood glycemic levels. These results also highlight the fast fluctuations and variability that can occur in refractive state, binocular and accommodative systems due to blood glycemia levels.

One interesting finding observed in the current study was the higher variance of values before lunch and insulin injection, which were highly reduced after lunch. This clinical case highlights the variations in the accommodative facility and accommodative amplitude by glycemia levels and points out the importance of optometric management in diabetic patients who come to the office for possible visual related symptomatology.

A lower efficacy of accommodative facility are linked to symptoms such as headaches, blur, or even diplopia [17]. Hence, patients with diabetes type I complaining visual symptoms are mandatory for optometrists or ophthalmologists to ask the levels of glycemia before any diagnosis or recommendations.

## 4. Conclusions

In this clinical case, we found intrasubject changes in myopic spherical refraction, and accommodative functions, specifically the amplitude of accommodation and accommodative facility, on low blood glucose levels. All levels returned to normal with appropriate blood glycemic levels. The myopic refraction decreased, the amplitude of accommodation increased, and the accommodation facility increased, nearly doubled, after caloric intake and restoration of normal blood sugar levels.

## Figures and Tables

**Table 1:** Median and standard deviation (SD) of refractive, binocular, and accommodative tests performed in the current study. “Pre” and “post” values are the mean and SD from 10 consecutive measures. The ranges from minimum to maximum values are shown in brackets below the mean and SD. On the right column are shown the *p* values from ANOVA. RE = Right Eye, LE = Left Eye, BI = Base In, BO = Base Out, NPC = Near Point of Convergence, AF = Accommodative Facility, AA = Amplitude of Accommodation, MEM = Lag of Accommodation.

		Pre	Post	*p* Value
Sphere (D *)	RE	−1.25 ± 0.39 [−1.75, −0.75]	−0.85 ± 0.26 [−1.50, −0.50]	*p* = 0.016
	LE	−1.50 ± 0.35 [−1.50, −0.50]	−1.22 ± 0.21 [−1.75, −1.00]	*p* = 0.051
Cylinder (D)	RE	−1.17 ± 1.35 [−1.00, −0.50]	−0.62 ± 1.31 [−0.75, −0.50]	*p* = 0.217
	LE	−0.85 ± 0.17 [−1.00, −0.50]	−0.80 ± 0.15 [−1.00, −0.50]	*p* = 0.511
BI far (Δ **)	B †	10.30 ± 2.66 [7.00, 16.00]	9.10 ± 2.13 [7.00, 14.00]	*p* = 0.281
	r ‡	6.60 ± 1.57 [4.00, 9.00]	6.00 ± 2.21 [4.00, 10.00]	*p* = 0.494
BI near (Δ)	B	18.10 ± 2.84 [12.00, 20.00]	19.80 ± 3.15 [16.00,25.00]	*p* = 0.222
	r	9.10 ± 6.96 [0.00, 18.00]	10.70 ± 6.14 [0.00, 17.00]	*p* = 0.593
BO far (Δ)	B	16.40 ± 3.92 [11.00, 20.00]	16.10 ± 4.25 [11.00, 22.00]	*p* = 0.872
	r	6.20 ± 2.57 [2.00, 10.00]	8.20 ± 3.32 [2.00, 12.00]	*p* = 0.150
BO near (Δ)	B	18.50 ± 7.24 [9.00, 13.00]	17.30 ± 4.59 [11.00, 24.00]	*p* = 0.664
	r	10.70 ± 10.04 [4.00, 24.00]	7.90 ± 6.96 [4.00, 16.00]	*p* = 0.478
NPC (cm)	Break	6.30 ± 0.67 [5.00, 7.00]	6.20 ± 0.63 [5.00, 7.00]	*p* = 0.736
	r	7.20 ± 0.78 [6.00, 8.00]	7.30 ± 0.67 [6.00, 8.00]	*p* = 0.764
AF (cpm)	RE	2.75 ± 3.67 [0.00–9.00]	6.90 ± 3.51 [0.00–11.00]	*p* = 0.019
	LE	3.90 ± 4.04 [0.00–10.00]	7.80 ± 3.22 [2.00–11.00]	*p* = 0.028
AA (D)	RE	9.85 ± 2.37 [6.75, 12.50]	11.35 ± 1.44 [8.50, 13.75]	*p* = 0.016
	LE	9.85 ± 12.02 [7.25, 13.00]	12.02 ± 1.21 [9.25, 13.00]	*p* = 0.019
MEM (D)	RE	0.50 ± 0.24 [0.00, 0.75]	0.32 ± 0.12 [0.25, 0.50]	*p* = 0.051
	LE	0.47 ± 0.22 [0.25, 0.75]	0.35 ± 0.13 [0.25, 0.50]	*p* = 0.137
Phoria (Δ)	Near #	1.60 ± 0.52 [1.00–2.00]	2.20 ± 0.78 [1.00–2.00]	*p* = 0.059
	Far	1.50 ± 0.53 [1.00–2.00]	1.50 ± 0.53 [1.00–4.00]	*p* > 0.100
Glucose (mg/dL)		176.10 ± 97.34 [52.00, 310.00]	106.30 ± 23.66 [61.00, 147.00]	*p* = 0.041

† = Break, ‡ = Recovery, * = Sphere Diopters, ** = Prism Diopters, # = Positives values indicates exophoria.

## Data Availability

The raw data supporting the conclusions of this article will be made available by the authors on request.
